# Reconstructing high-resolution chromosome three-dimensional structures by Hi-C complex networks

**DOI:** 10.1186/s12859-018-2464-z

**Published:** 2018-12-28

**Authors:** Tong Liu, Zheng Wang

**Affiliations:** Department of Computer Science, University of Miami, 1365 Memorial Drive, Coral Gables, FL 33124 USA

**Keywords:** Chromosomal three-dimensional structure, Hi-C complex network, Wish distance, Converting parameter, Small-world network, Topologically associating domain

## Abstract

**Background:**

Hi-C data have been widely used to reconstruct chromosomal three-dimensional (3D) structures. One of the key limitations of Hi-C is the unclear relationship between spatial distance and the number of Hi-C contacts. Many methods used a fixed parameter when converting the number of Hi-C contacts to wish distances. However, a single parameter cannot properly explain the relationship between wish distances and genomic distances or the  locations of topologically associating domains (TADs).

**Results:**

We have addressed one of the key issues of using Hi-C data, that is, the unclear relationship between spatial distances and the number of Hi-C contacts, which is crucial to understand significant biological functions, such as the enhancer-promoter interactions. Specifically, we developed a new method to infer this converting parameter and pairwise Euclidean distances based on the topology of the Hi-C complex network (HiCNet). The inferred distances were modeled by clustering coefficient and multiple other types of constraints. We found that our inferred distances between bead-pairs within the same TAD were apparently smaller than those distances between bead-pairs from different TADs. Our inferred distances had a higher correlation with fluorescence in situ hybridization (FISH) data, fitted the localization patterns of Xist transcripts on DNA, and better matched 156 pairs of protein-enabled long-range chromatin interactions detected by ChIA-PET. Using the inferred distances and another round of optimization, we further reconstructed 40 kb high-resolution 3D chromosomal structures of mouse male ES cells. The high-resolution structures successfully illustrate TADs and DNA loops (peaks in Hi-C contact heatmaps) that usually indicate enhancer-promoter interactions.

**Conclusions:**

We developed a novel method to infer the wish distances between DNA bead-pairs from Hi-C contacts. High-resolution 3D structures of chromosomes were built based on the newly-inferred wish distances. This whole process has been implemented as a tool named HiCNet, which is publicly available at http://dna.cs.miami.edu/HiCNet/.

**Electronic supplementary material:**

The online version of this article (10.1186/s12859-018-2464-z) contains supplementary material, which is available to authorized users.

## Background

The chromosome conformation capture techniques [1–4] can detect physical interactions between a pair of genome loci. Especially, the recent Hi-C technique [5] can identify chromosome contacts at the whole genome level. In the past few years, Hi-C experiments have been conducted on different species and cell lines [5–9]; and the resolution of Hi-C experiments keeps increasing from 1 Mb to 1 kb [6, 9]. Recently, a computational method that uses deep learning has been developed to enhance Hi-C data resolution [10].

Hi-C contact data have been widely used in different fields, such as exploring Xist transcript mechanism [11], predicting DNA methylation [12], and revealing structural properties of chromosomes, e.g., topologically associating domains (TADs) [6] and peaks/loops [9]. Topologically associating domains (TADs), a segment of a chromosome with megabase size or smaller, have been found to be conserved between different cell lines and across different species [6]. TADs are identified based on the property that the Hi-C contact counts within a TAD are apparently higher than those between two adjacent TADs. It has also been tested that the boundary regions of TADs are enriched with some genomic factors [6], such as insulator binding protein CTCF. Loops are identified from local peaks in a Hi-C contact matrix: the peak pixels have an apparent enrichment of Hi-C data, while the pixels in their neighbourhood do not seem to have high contact counts. A peak indicates that there may be a loop physically residing in the peak region. Peaks are also conserved across different cell lines and species and can reside in topological domain boundaries and CTCF binding sites [9]. However, it has been proved that there are some systematic biases in raw Hi-C data [13, 14]. Therefore, before using Hi-C data we need to remove these biases. There are some efficient normalization tools for eliminating the known biases (e.g., restriction enzyme cutting sites, GC content, and mappability) in raw Hi-C data, such as Hicpipe [13], ICE [15], HiCNorm [16], KR [9, 17], and scHiCNorm [14].

Another important application of Hi-C data is to reconstruct chromosome 3D structures. Several methods based on simulation and probability models have been developed [18–24]. A widely created method is to first convert Hi-C contacts into wish Euclidean distances based on the assumption that wish distances follow power law distribution with Hi-C contacts (*δ = c*^*-α*^, *δ*: wish distance, *c*: Hi-C contact number, and *α*: a converting parameter) and then followed by an optimization process that calculates three-dimensional coordinates using algorithms such as metric multidimensional scaling [21, 22, 24].

It has been observed that Hi-C contact probability of mammalian chromosomes is inversely proportional to genomic distance on each chromosome [5] (*c* ~ *s*^− 1^). Meanwhile, based on previous studies of polymers the volume scales are proportional to the chain length (*d*^3^ ~ *s*) (e.g. genomic distance) [25]. Therefore, Varoquaux et al. [21] concluded that the relationship between Hi-C contacts and spatial distances was *d* ~ *c*^-1/3^ (i.e., α = 1/3). Based on this conclusion, they modeled chromosomal 3D structures at different resolutions using the same parameter (1/3). However, this arbitrary converting between number of Hi-C contacts and wish distances has drawbacks, especially when applied to different resolutions [22], different organisms [21, 26], and different time points during cell cycle [27]. For cases when number of Hi-C contacts are larger than 10, the converted wish distances using *δ* ~ *c*^-1/3^ are very small and almost have no difference (Additional file 1: Figure S1a), which makes it hard to distinguish these interactions in terms of spatial distance. For example, for the contacts between positions with 20 beads apart, (a chromosome is evenly divided into beads; and each bead is 40 kb), in today’s high-resolution Hi-C data sets > 50% of them have the number of Hi-C contacts larger than 10 (Additional file 1: Figure S1b). This indicates that the *δ* ~ *c*^-1/3^ formula may not work well nowadays when the Hi-C experiments can reach a high resolution by generating significantly larger number of Hi-C reads.

Therefore, it is reasonable to assume *δ* ~ *c*^-α^; but α should be bead-pair dependent instead of a fixed value for all bead-pairs. Zhang et al. [22] designed a method to dynamically assign values for α, which used semi-definite embedding to infer the spatial organizations of chromosomes and then calculated Hi-C reversely to obtain the optimal α in which the inferred Hi-C contacts best fitted the original ones. The whole process was time-consuming as it needed to reconstruct the 3D structure at the beginning. In comparison, our method does not need to generate a 3D structure first. Chromosome3D [24] used the Spearman correlation between Hi-C contact and inferred distances to tune the parameter, but it still needed to generate many structures to obtain the best parameter.

In order to evaluate the reconstructed 3D structure, the distances parsed from the reconstructed 3D structure are usually compared with fluorescence in situ hybridization (FISH) data [6, 19, 20]. The chromosomal interactions detected by FISH are usually considered accurate, and therefore used as benchmarks. However, it is in a small scale because usually only a couple of genomic interactions can be detected by FISH. Therefore, we also used the Xist localization intensity on X-chromosome and ChIA-PET to evaluate our structures.

Engreitz et al. [11] conducted RNA Anti-sense Purification (RAP) experiments in mouse embryonic stem (ES) cells to detect the localization intensities of lncRNA Xist when X-chromosome was being inactivated. They found that Xist transcripts more intensively bound at the DNA sites in spatial proximity to the Xist locus but less intensively on the DNA sites spatially far away from the Xist locus (Hi-C contact data were used to measure spatial proximity). They detected a significant correlation between 3D distances to Xist locus and the Xist localization intensities. If the inferred distances or inferred 3D structures make sense, the same strong correlation should be found.

Dowen et al. [28] have applied cohesion ChIA-PET in mouse ES cells to detect protein-enabled long-range chromatin interactions. An unique feature of ChIA-PET is the inclusion of chromatin immunoprecipitation (ChIP) at the beginning to enrich the fragments bound by a particular protein of interest [29]. Together with the design of using two aliquots before fragment ligation, these make ChIA-PET good at detecting protein-enabled interactions [29]. Therefore, we can use these ChIA-PET-confirmed interactions to evaluate our inferred Euclidean distances or reconstructed 3D structures.

In this study, we present a new method to model the converting factor α based on the tendency of a bead to be clustered with neighboring beads in a complex network named Hi-C network (HiCNet). The optimized converting factor α enables us to directly generate optimized pairwise Euclidean distances without generating a 3D structure. The optimized distances are not only consistent with the definitions of intra- and inter-TADs, but also well fit FISH data and ChIA-PET confirmed interactions. We further used the optimized distances and another round of optimization to reconstruct the chromosomal 3D structures of mouse ES cells at 40 kb high resolution and found that compared to other existent methods our inferred 3D structures better fit a FISH data set.

## Methods

The input of our method is a normalized [13] Hi-C contact matrix ***C*** at a high resolution, e.g., 40 kb. The matrix is symmetric; and each row or column corresponds to a fixed bead size (e.g. 40 kb). The target in this step is to generate an optimized distance *δ*_*ij*_ for each Hi-C value *c*_*ij*_ in ***C***. The relationship between *δ*_*ij*_ and *c*_*ij*_ follows the power law distribution as shown in Eq. 1:

1 $$ {\delta}_{ij}={c_{ij}}^{-{\alpha}_{ij}}\mathrm{if}\ {c}_{ij}>0 $$

**Fig. 1:**
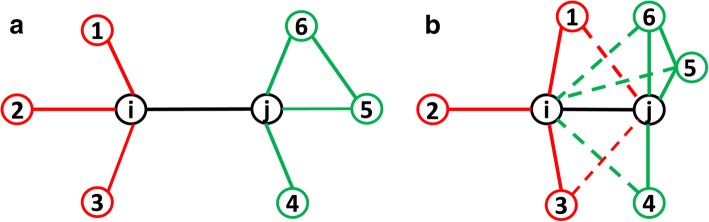
**a** An illustration of Hi-C contacts between two beads *i* and *j* with smaller α_HiCNet_ parameter (longer wish distance). Notice that there is always an edge connecting nodes *i* and *j* as we only model the bead pairs that have non-zero number of Hi-C contacts. Therefore, beads *i* and *j* are immediate neighbors of each other. **b** An illustration of Hi-C contacts between two beads *i* and *j* with larger α_HiCNet_ parameters (smaller wish distance). Using node *j* as an example, previously there was no edge connecting nodes 5 and *i*, both of which are the immediate neighbors of *j*. However, in **b** nodes 5 and *i* are connected; nodes 4 and *i* are connected; and nodes 6 and *i* are connected. This results in a higher clustering coefficient for node *j*, similarly if observing from the perspective of node *i* (nodes *j* and 1 are connected; and nodes *j* and 3 are connected, increasing the clustering coefficient of node *i*). Therefore, both nodes *i* and *j* will have a higher tendency to be closer in case **b** compared with case **a**

Notice that every pair of beads has a specifically-optimized factor *α*_*ij*_. This is different from a previous work [21], in which a fixed α is used for all bead-pairs. Specifically, *α*_*ij*_ is calculated as

2 $$ {\alpha}_{ij}=\left({w}_i{\alpha}_i+{w}_j{\alpha}_j\right)/\left({w}_i+{w}_j\right) $$

in which α_*i*_ and α_*j*_ are the “clustering strength” of beads *i* and *j*, a new term we define to measure a bead’s tendency of being clustered with the neighboring beads on the same chromosome. The *w*_*i*_ and *w*_*j*_ are the sum of Hi-C contacts in the *i*th and *j*th rows of normalized Hi-C matrix ***C***, respectively. In this way, the parameter α_*ij*_ is determined by the “clustering strength” of both beads *i* and *j* but normalized by the Hi-C contacts related to beads *i* and *j*. The heuristic is that if two beads both have higher tendencies of being clustered with neighboring beads, their α_*ij*_ value should be relatively higher, which makes their wish distances relatively smaller. Figure 1 illustrates this heuristic using two examples. The “clustering strength” of the bead with higher Hi-C value will have larger weight in the bead pair. The proof with real data can be found in Results section.

To model the “clustering strength” of a bead, we introduced a novel type of complex network, in which every vertex represents a 40 kb bead; and if the Hi-C contacts between two beads are not zero, an edge is created to connect the two corresponding vertices. This is different to our previous research [30] as it changes the meaning of vertices from genes to beads with a higher resolution. The clustering coefficient of a vertex in the complex network is used to model the “clustering strength” of a bead/vertex:

3 $$ {CC}_i=\frac{2{e}_i}{K_i\left({K}_i-1\right)} $$

where *e*_*i*_ is the number of connected vertex pairs among immediate (one edge away) neighboring vertices of the target vertex *i*; and *K*_*i*_ is the number of immediate neighboring vertices of the target vertex *i* [31].

However, we cannot arbitrarily set each bead’s “clustering strength” as its clustering coefficient in the complex network because all beads form up a complex system and the final value of every bead’s “clustering strength” must be set in a way that the global system is optimized. Therefore, we used clustering coefficient as the target value and performed an optimization using the following objective function:

4 $$ argmin\_{\sum}_{i= 1}^n{\lambda}_1{\left({a}_i\hbox{-} {\lambda}_2{CC}_i\right)}^2+{\sum}_{\left(i,j,k\right)\in PT}{\lambda}_3\left\{{\left({a}_i\hbox{-} {a}_k\right)}^2+{\left({a}_j\hbox{-} {a}_k\right)}^2\right\} $$

where the first part of the formula (before the first plus sign) tries to assign “clustering strength” for every bead with clustering coefficient as the target value.

The second part (after the first plus sign) in Eq. (4) is related to a set *PT*, which contains all the triples consisting of bead *i*, bead *j*, bead *k*, where

5 $$ PT=\left\{\left(i,j,k\right)|{p}_{ij}>{p}_0,{p}_{ik}>{p}_0,{p}_{jk}>{p}_0\right\} $$

In this equation, *p*_*ij*_ is the Pearson’s correlation coefficient between the *i*th row and *j*th row in the normalized Hi-C matrix, which are the Hi-C profiles between the *i*th and *j*th beads with all other beads, respectively. Therefore, a high value *p*_*ij*_ indicates that the *i*th and *j*th beads are spatially close because these two beads have similar Hi-C contact patterns with all other beads. In Eq. (5), *p*_*0*_ is a threshold and is set to 0.95 in our research. In this way, the second term of Eq. (4) tries to achieve this: if any two beads in a triple have a high correlation (e.g., > 0.95), their “clustering strength” values *α*_*i*_, *α*_*j*_, and *α*_*k*_ should be highly similar or the same. These triples put important global constraints to the inferred “clustering strength” because the three beads in the triples may not be adjacent but irregularly spread over the entire chromosome. Multiple triples like that can improve the accuracy of inferred distances as it adds the consideration of correlations on normalized Hi-C contacts, which have been found helpful to remove noise from raw Hi-C contact matrices [30].

The λ values (i.e., λ_1_, λ_2_, and λ_3_) in Eq. (4) are weight parameters tuned based on fluorescence in situ hybridization (FISH) data (six pairs, three from chromosome 2 and the other three from chromosome 11) from [32].

Eq. (4) is also subjected to the following two constraints:

6 $$ 0\le {\alpha}_i\le 1i\in \left[1\cdots n\right] $$

7 $$ \left\{\begin{array}{c}{\delta}_{ij}+{\delta}_{ik}\ge {\delta}_{jk}\\ {}{\delta}_{ij}+{\delta}_{jk}\ge {\delta}_{ik}\\ {}{\delta}_{ik}+{\delta}_{jk}\ge {\delta}_{ij}\end{array}|\begin{array}{c}\ \left|i-j\right|=1,k\ne i,k\ne j,\\ {}{c}_{ij}>0,{c}_{ik}>0,{c}_{jk}>0\end{array}\right\} $$

The second constraint is the triangle inequality, where *δ*_*ij*_ is the inferred distance between beads *i* and *j*. It can be found that this constraint contains a large number of triangles consisting of triple beads (Additional file 1: Figure S1c). This tries to make the inferred distances δ between the three beads not violating triangle inequality. These triangles have a regular pattern (*i* and *j* are adjacent; and *k* cannot be *i* or *j*) and more densely exist on the chromosome, which is different from the triples in Eq. (5). They both constrain the inferred distances but from different perspectives.

Notice that by solving the above optimization problem, we get the inferred distances *δ*_*ij*_, which is the optimized Euclidean distances between every pair of beads. For many studies, these optimized distances are all we need, such as calculating the correlation between Euclidean distances and Xist localization intensities [11]. To many studies, the final purpose of reconstructing a 3D structure is to analyze it in a quantitative way; and the pairwise Euclidean distances are one of the most frequently used structural features of a 3D structure.

We also assigned the inferred distances back to the Hi-C complex network as the weight of edges. In this way, the weighted Hi-C complex networks can directly provide optimized Euclidean distance for all bead pairs with no need to reconstruct the 3D structure.

If needed, based on the already optimized distances *δ*_*ij*_, we still can reconstruct the 3D structure for visualization. We applied another round of optimization using metric multidimensional scaling (MDS):

8 $$ argmin{\sum}_{c_{ij}>0}\frac{{\left({d}_{ij}-{\delta}_{ij}\right)}^2}{{\delta_{ij}}^2}+\sum \limits_{c_{ij}=0}\frac{{\left({d}_{ij}-R\right)}^2}{R^2} $$

where *d*_*ij*_ was the Euclidean distance between beads *i* and *j* in the reconstructed 3D structure; *R* was used to limit the distance between two beads when their number of Hi-C contact equals zero (i.e, *c*_*ij*_ = 0). In this study, *R* was set to the maximum wish distances in the weighted Hi-C complex network. The metric multidimensional scaling algorithm tries to rearrange the 3D coordinate of every bead to make the Euclidean distances *d*_*ij*_ close to the optimized distances *δ*_*ij*_. Because the target distances *δ*_*ij*_ have already been optimized under multiple types of constraints, no constraint is needed here, which makes this process quick. The two optimization problems in Eqs. 4 and 8 were solved by IPOPT [33].

## Results

### Relationships between inferred distances and Hi-C contacts

The normalized Hi-C data were downloaded from http://chromosome.sdsc.edu/mouse/hi-c/download.html. Our method was performed on 20 chromosomes of mouse embryonic stem (ES) cells at the resolution of 40 kb. The distribution of optimal α parameters for the twenty chromosomes can be found in Additional file 1: Figure S2.

**Fig. 2:**
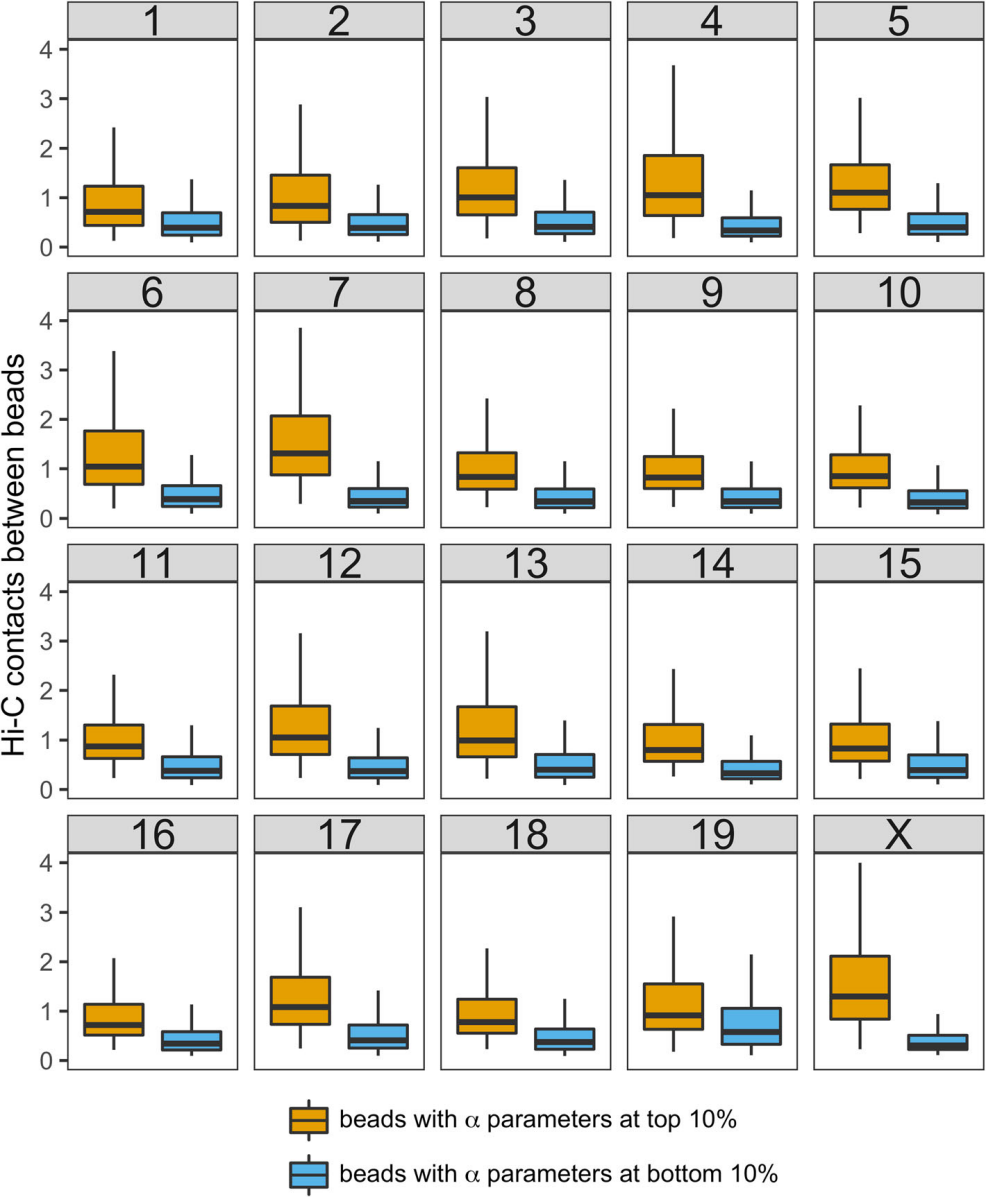
The distribution of Hi-C contacts between the beads with α parameters at top 10% and between the beads with α parameters at bottom 10%

First, we need to confirm that two beads with larger α_HiCNet_ parameters correspond to higher Hi-C contacts, which result in smaller wish distances. For each chromosome, we extracted beads with α_HiCNet_ parameters at top 10% and plotted the distribution of Hi-C contacts between these beads; we did the same work for beads with α_HiCNet_ parameters at bottom 10%. The results shown in Fig. 2 indicate that two beads with larger α values (caused by higher clustering coefficients) have more Hi-C contacts, which result in smaller wish distances. We can draw the same conclusion if we vary the top-bottom level (for 5% see Additional file 1: Figure S3, and 20% see Additional file 1: Figure S4). These observations explain our assumption that two beads with larger clustering coefficients have much more enriched Hi-C contacts than two beads with smaller clustering coefficients.

Second, we also found that α_*ij*_ is positively correlated with Hi-C contact *c*_*ij*_ (see Additional file 1: Figure S5) when we only considered Hi-C contacts not equal to zero and genomic distance between two beads (i.e., |*i* - *j*|) larger than 0.1 times total number of beads on a chromosome, which was following the same practice as in [24].

**Fig. 3:**
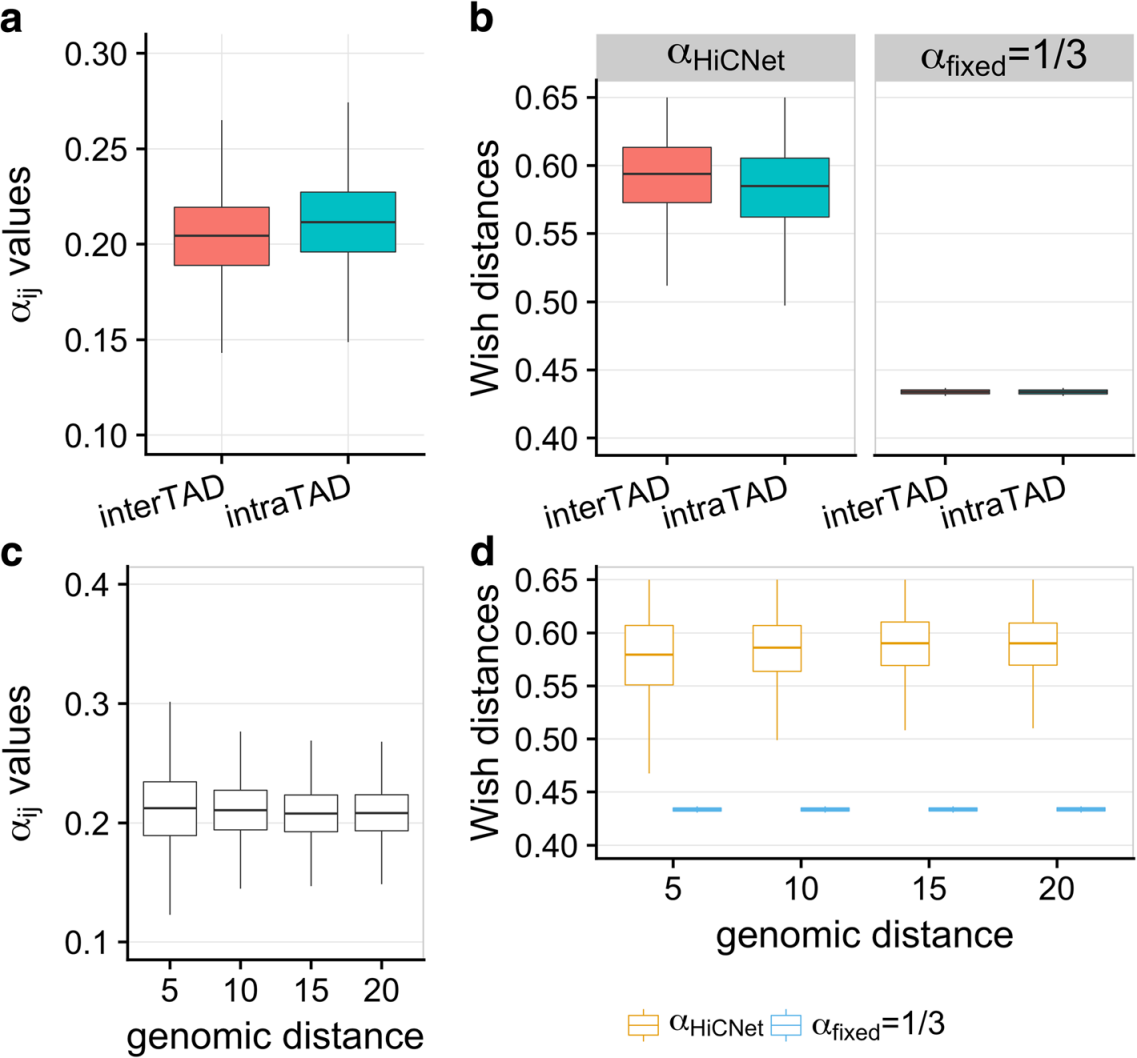
**a** The distribution of α_*ij*_ values when the two beads are within the same and different TADs for bead pairs with Hi-C contacts in the range [12, 12.5]. **b** The distribution of wish distances inferred from HiCNet and α = 1/3 when the two beads are within the same and different TADs for bead pairs with Hi-C contacts in the range [12, 12.5]. **c** and **d** The distribution of α_*ij*_ values and wish distances for different genomic distance intervals (i.e, [1, 5], (5, 10], (10, 15], (15, 20]) for bead pairs with Hi-C contacts in the range [12, 12.5]. Here the genomic distance means the number of beads (each bead with 40,000 bp) away

Third, we explored the relationships between α_*ij*_ and TADs. Here, TADs’ locations were called using domaincaller [6]. We next extracted all bead pairs with the number of Hi-C contacts in a small range [12, 12.5], which resulted in 29,752 bead pairs. We assigned intra-TAD or inter-TAD for each bead pair based on whether two beads were within the same TAD. From the definition of TADs, we expected that intra-TAD bead pairs have larger α_*ij*_ values than inter-TAD pairs when the Hi-C contacts were within the same small range, i.e., [12, 12.5]. Fig. 3 shows that intra-TAD bead pairs have larger α_*ij*_ values that correspond to smaller wish distances, but if we have used a fixed value (i.e., α = 1/3) we cannot distinguish the wish distance differences between intra- and inter-TADs. Figure 3 also shows that with the increase of genomic distances the α_*ij*_ values decrease; the wish distances from HiCNet are more distinguishable than those from α = 1/3.

### Small-world properties of Hi-C complex networks

We constructed the Hi-C complex network for each chromosome, e.g., the Hi-C network for chromosome 10 had 3164 vertices and 9492 edges; and the Hi-C network for X-chromosome had 3651 vertices and 10,953 edges.

**Fig. 4:**
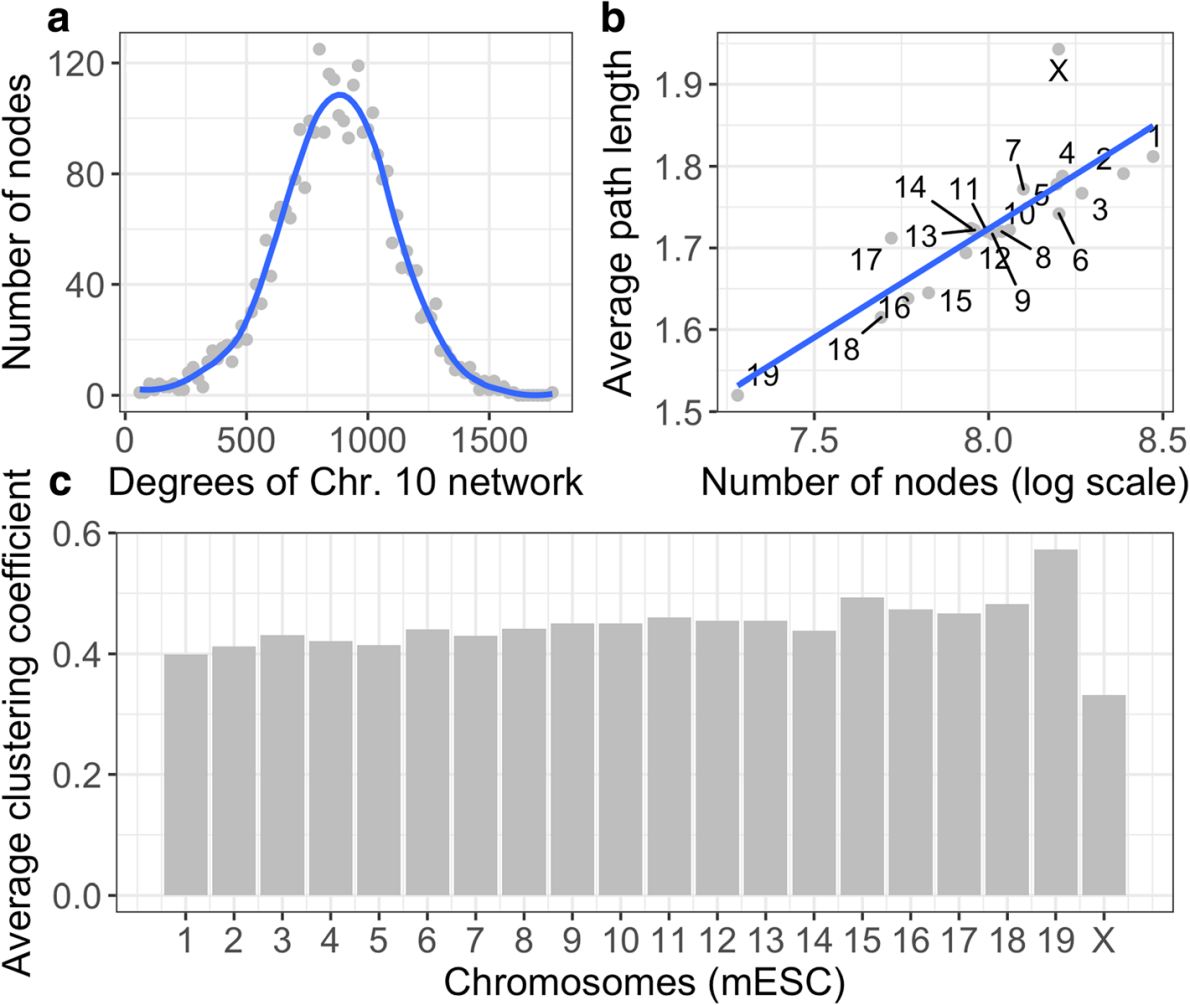
**a** The distribution of node degrees for chromosome 10 network; **b** The plot of number of nodes in each chromosome network against the average path length; **c** The average clustering coefficients for the 20 chromosome networks in mESC

We explored whether Hi-C complex networks belonged to one of the two most common types of complex networks: scale-free networks and small-world networks. As for scale-free networks, the degree distribution follows a power law, indicating that a smaller number of high-degree nodes have an important role in the network. However, the degree distribution of Hi-C complex networks does not follow a power law; and most of nodes have an average number of degrees (Fig. 4a).

A small-world network [34] is defined as having the following properties: (1) a small average shortest path length L; (2) a large clustering coefficient; (3) the average path length L is proportional to the logarithm of the number of nodes in the network. The 20 networks we have created for mESC meet all three properties: (1) the average path lengths of 20 chromosome networks are within [1.5, 2.0] (Fig. 4b); (2) the average clustering coefficients for the 20 chromosome networks are mostly within [0.4, 0.6] (Fig. 4c); (3) with the increase of the logarithm of the number of vertices in each network, the average path length grows proportionally (Fig. 4b). There are two chromosomes that are particularly interesting: chromosome 19 that has the smallest path length but has the largest average clustering coefficient and X-chromosome that has the largest path length but has the smallest average clustering coefficient. Future research can be conducted to further study their network topologies.

### Evaluation of the inferred distances by FISH, RAP, and ChIA-PET

First, we compared our inferred distances with FISH data (six pairs, three from chromosome 2 and the other three from chromosome 11) from [32] in mouse embryonic stem (ES) cells. Because parameters in the target function (Eq. 4) were tuned based on this FISH data, it was not surprising to see that our inferred distances achieve a higher correlation with the FISH data (*r* = 0.81) compared to α_fixed_ (*r* = 0.73). Both are better than randomly selected α values (*r* = 0.59).

Second, we used the localization intensities of a long non-coding RNA Xist to evaluate our inferred distances. Engreitz et al. [11] found that Xist transcripts are more intensively bound to those DNA sites in spatial proximity to Xist locus but less intensively to the DNA sites that were far away from Xist locus (significant correlations found). We used RAP data to see whether our inferred distances matched this finding. Our method outperformed *α*_*fixed*_ by a higher correlation with RAP data (*r* = − 0.64, *n* = 906) than *α*_*fixed*_ (*r* = − 0.59); and both are better than random α values (*r* = − 0.36).

**Fig. 5:**
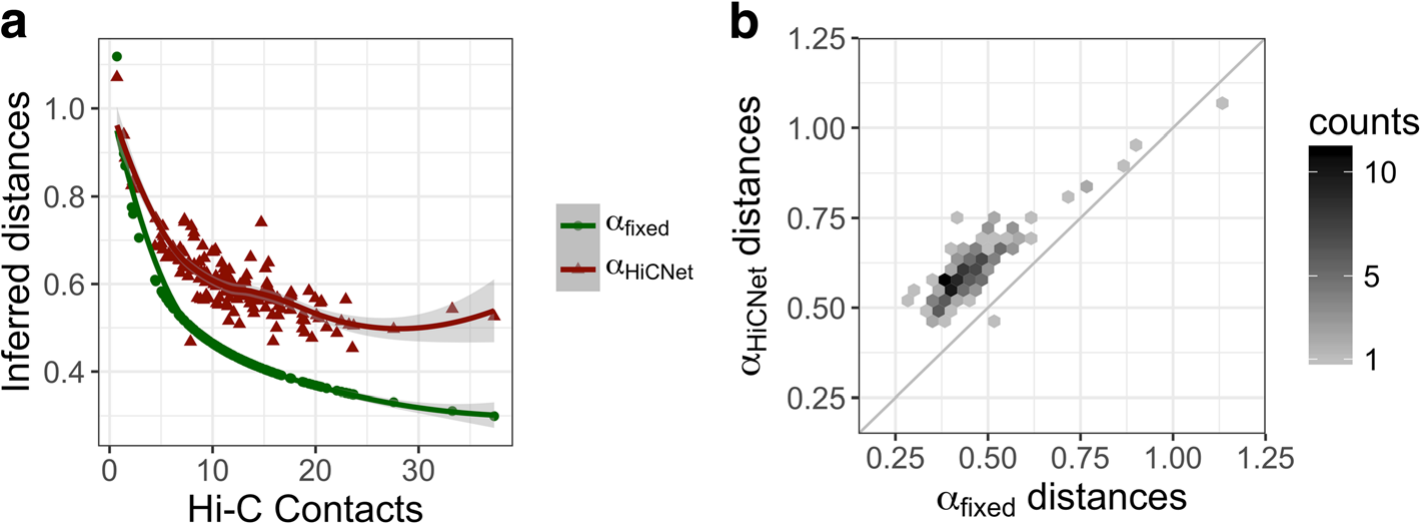
**a** The distance distribution for two methods to determine α values (α_fixed_ = 1/3 and α_HiCNet_) corresponding to different Hi-C contacts; **b** The plot of the α_fixed_ distances against the α_HiCNet_ distances

Third, we downloaded ChIA-PET dataset consisting of 23,835 protein-enabled chromatin interactions [28]. We performed a filtering process that only kept the long-range interactions with sequential distance larger than or equal to 25 beads (each bead is 40 kb), resulting in 163 pairs. After excluding the contacts for which optimized distance could not be inferred because of missing Hi-C values, we finally obtained 156 bead-pairs. The ideal outcome would be that all the 156 ChIA-PET interacting beads were having the same or highly similar Hi-C inferred wish distances because these interactions were all formed by the same biological mechanism, that is, protein-protein interaction [28]. However, Fig. 5 shows that the Hi-C inferred distances using α_fixed_ (i.e., 1/3) are more scattered (spans three grids) compared to the distances obtained by our α_HiCNet,_ which mostly vary within [0.5, 0.7] (Fig. 5a) and span two grids (Fig. 5b). Additional file 1: Figure S6 shows the relationship between HiCNet-inferred distances and Hi-C contacts for chromosome 9 with Hi-C contact <=50. These observations suggest that our inferred distances better fit the protein-enabled long-range interactions captured by ChIA-PET_._

### Chromosomal 3D structure inference using Hi-C complex networks

**Fig. 6:**
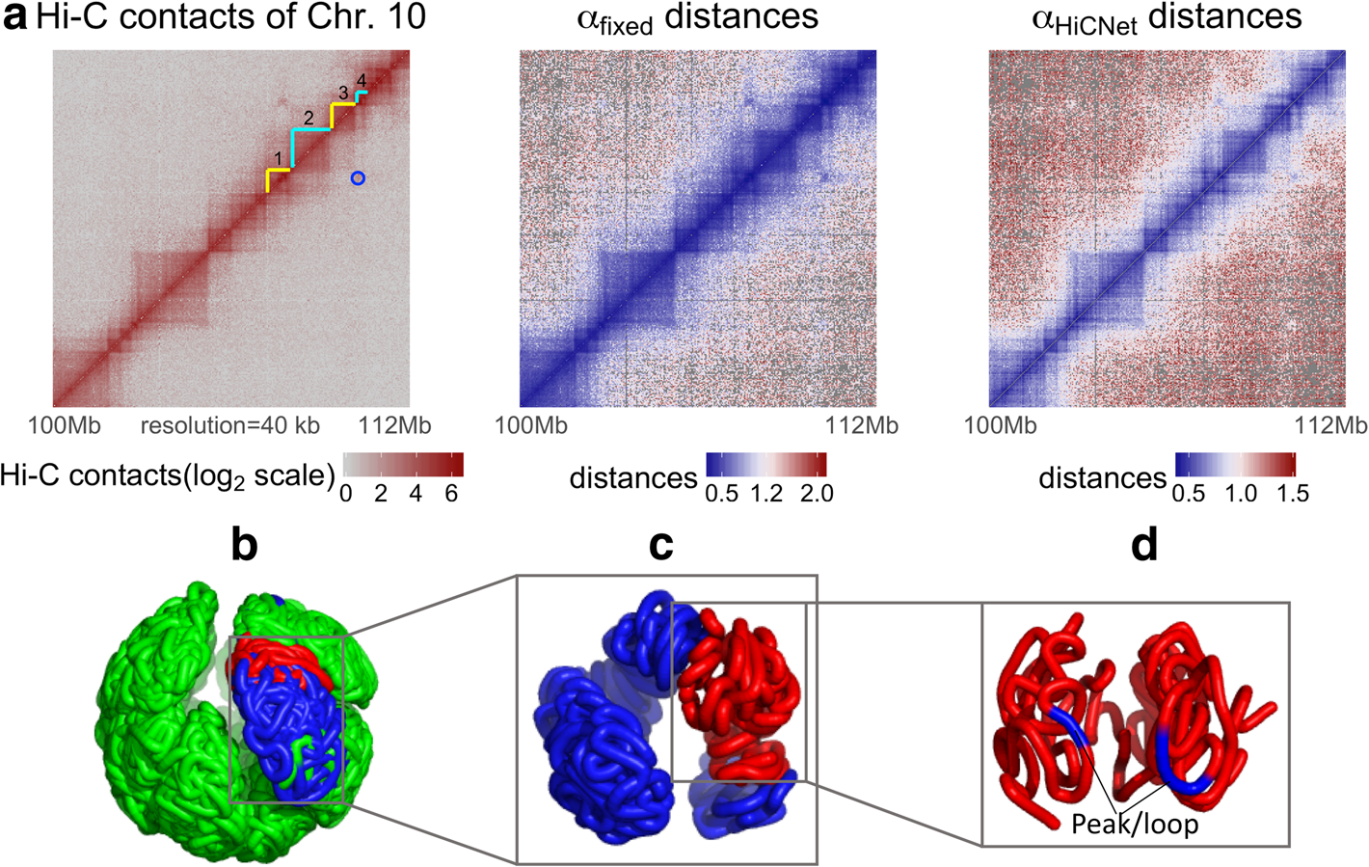
**a** The two-dimensional heat map of Hi-C contacts of chromosome 10 in part (one peak marked with a blue circle and four topological domains around the peak with numbers labelled); and the heat maps of α_fixed_ inferred and α_HiCNet_ inferred distances. **b** The three-dimensional structure of chromosome 10 (resolution = 40 kb). **c** The three-dimensional structure of chromosome 10 in part (i.e., 100 Mb – 112 Mb) corresponding to the part in **a**. **d** The three-dimensional structure of the four topological domains highlighted in **a**

Based on the optimized distances, we reconstructed the 3D structures of all mouse ES cell chromosomes. We visualized the Hi-C contact heatmap and wish distances heatmaps (both α_fixed_ and α_HiCNet_) of a segment of chromosome 10 (i.e., 100 Mb – 112 Mb), in which there are about 12 TADs and one peak/loop (Fig. 6a). Notice that the peak usually indicates enhancer-promoter interaction. The corresponding inferred distances are shown in Fig. 6a for α_fixed_ and α_HiCNet_, respectively. Both can indicate TAD patterns, but the boundaries of TADs using α_HiCNet_ are much clearer and sharper compared to the ones using α_fixed_. This indicates that our method can better distinguish the beads in the domain boundary regions. We also present the 40 kb high-resolution 3D structure of the entire chromosome 10 (Fig. 6b), zoomed-in chromosome 10 in part (Fig. 6c), and further zoomed-in plot showing four TADs (Fig. 6d). Notice that the peak highlighted in the 3D structure of Fig. 6d is corresponding to the peak highlighted in the Hi-C heatmap in Fig. 6a (the blue circle). These high-resolution structures allow us to clearly illustrate how DNA loop is formed.

**Fig. 7:**
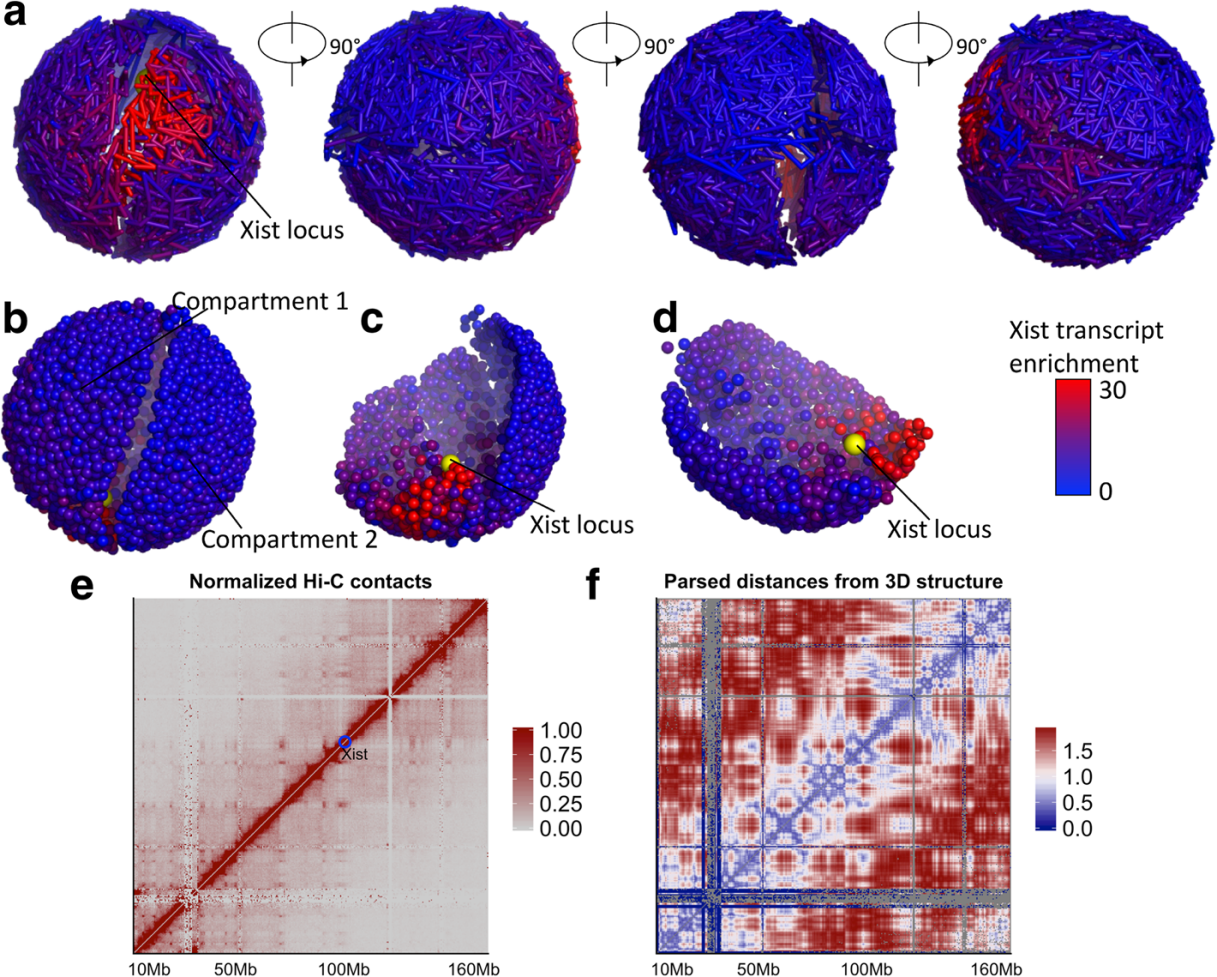
**a** The 3D structure of X-chromosome with Xist transcript enrichment mapped. **b** The 3D structure of X-chromosome (without edges) consisting of two compartments. **c** and **d** The interior structures of compartment 1 and compartment 2 respectively. **e** The normalized Hi-C heatmap of X-chromosome at the resolution of 500 kb (the blue circle highlights the Xist locus). **f** The 500 kb resolution distance heatmap of X-chromosome parsed from the reconstructed 3D structure with 40 kb resolution

We also modeled the 3D structure of X-chromosome with Xist transcript localization intensities (after one hour of generating Xist transcripts) mapped onto the 3D structure, as shown in Fig. 7. The high-resolution structure clearly shows that the X-chromosome has two separate compartments as shown in Fig. 7b. This matches the finding from another research [11], that is, X-chromosome contains two mega-domains separated by a boundary region. We also high-lighted lncRNA Xist locus (Fig. 7c and d). Moreover, from the figure we can observe that the regions surrounding the Xist locus are more enriched with Xist transcripts, whereas the regions further away from the Xist locus have less Xist transcript localizations.

We further tested whether our inferred 3D structures fitted Hi-C contact patterns. We generated a Hi-C contact heatmap of X-chromosome at the resolution of 500 kb, which was normalized by KR method (Fig. 7e). Plotting the heatmap for the whole chromosome at 40 kb resolution is hard to achieve. However, we did plot 40 kb resolution heatmaps for a segment of chromosome 10 (see Fig. 6). We then parsed the Euclidean distances from the reconstructed 40 kb resolution 3D structure and averaged them into 500 kb resolution. In this way, we were able to draw the distance heatmap at 500 kb resolution (Fig. 7f). We performed the same procedure and plotted the heatmaps of distances parsed from the 40 kb resolution 3D structures generated by PASTIS (Additional file 1: Figure S7) and ChromSDE (Additional file 1: Figure S8). From Fig. 7e and f, we observed that our inferred 3D structure better matched the general patterns in Hi-C contact heatmap.

**Fig. 8:**
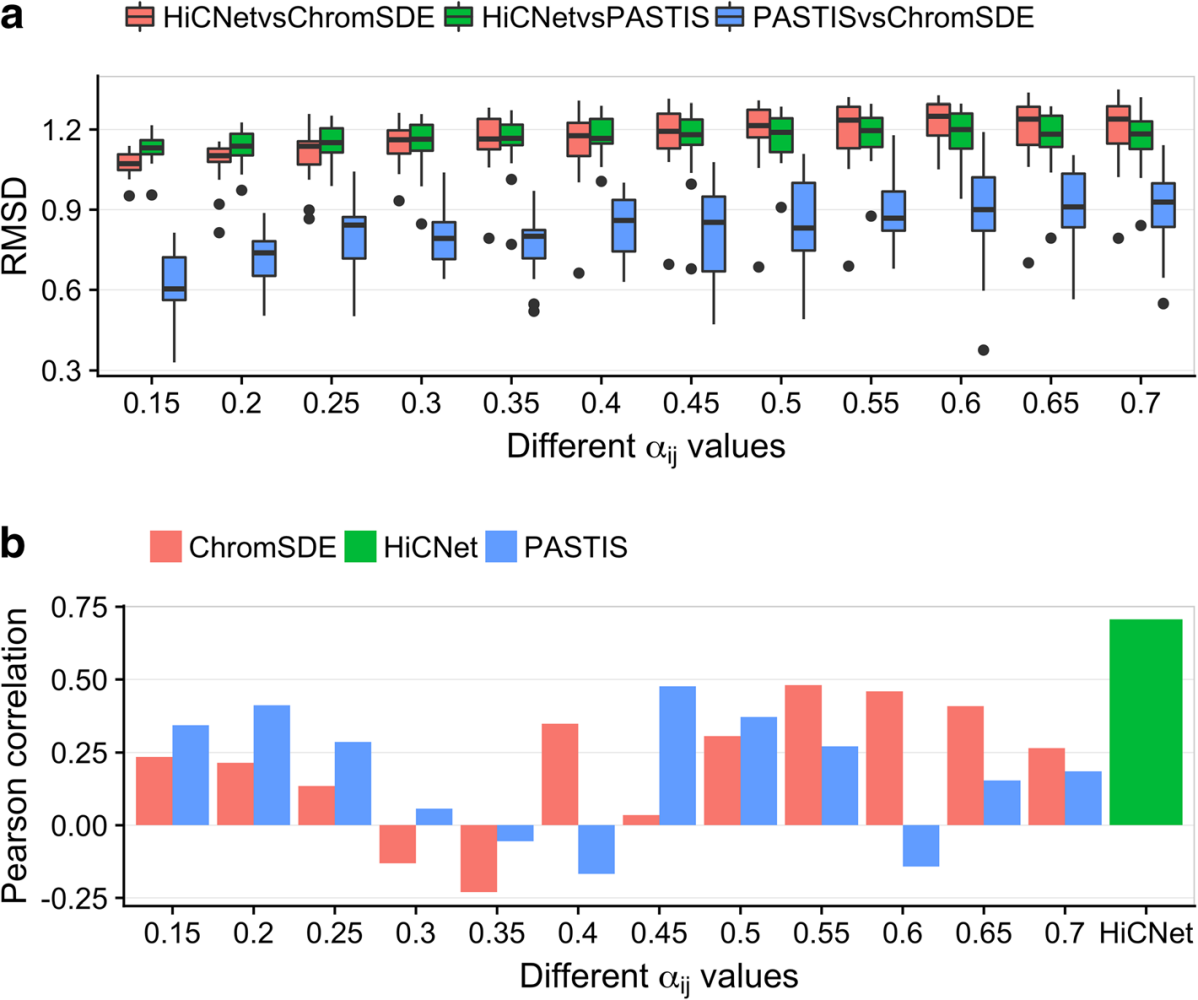
**a** The Root Mean Squared Deviations between any two 3D structures from HiCNet, PASTIS, and ChromSDE when we choose different α values for PASTIS and ChromSDE. **b** The Pearson’s correlation coefficients between a new FISH data set and the parsed average distances from the 3D structures constructed by HiCNet, PASTIS and ChromSDE (with different α values)

We next compared the 3D structures we inferred with those inferred from PASTIS (MDS) [21] and ChromSDE [22]. We used different α values for PASTIS and ChromSDE, but the optimal solutions were all obtained using IPOPT [33] for direct comparison between different 3D structures. We used the Kabsch algorithm [35] to minimize the root mean squared deviation (RMSD) between two 3D structures. The results are shown in Fig. 8a, indicating that the 3D structures from HiCNet are slightly different to those from PASTIS and ChromSDE, which is reasonable because HiCNet assigns a distinct α value for each pair of beads, whereas PASTIS and ChromSDE only use a single α value for all bead pairs, resulting in different wish distance distributions between HiCNet and the other two methods. Moreover, we used another FISH data set [36] (eight pairs of median values, four pairs from chromosome 3, and four pairs from chromosome 11) to determine which methods’ results are more consistent with the new FISH data. Our average distances parsed from 3D structures have a higher Pearson’s correlation than those from PASTIS and ChromSDE with α equal to different values (see Fig. 8b). Because the FISH experiment was conducted at the resolution of 500 kb and our 3D structures were reconstructed at the resolution of 40 kb, here for each pair of FISH data set (i.e., two segments on a chromosome, each with 500,000 bp) the average distance parsed from 3D structures was the average value of all parsed distances between any two beads found in the two different segments.

## Discussion

There are many studies that can reconstruct chromosomal 3D structures. However, the goal of reconstructing chromosomal 3D structures is not only to visualize the structure, but also to quantitively analyze the 3D structures. For many cases, the Euclidean distances between all bead pairs are the only information needed for the quantitative analysis on a 3D structure. In this type of analysis, our optimized distances can directly be used with no need to reconstruct a 3D structure (and then parse the distances out from the 3D structure).

Moreover, after we assign the optimized distances as the weights of edges back to the Hi-C complex networks, the topology of this type of networks has integrated optimized Euclidean distances in the 3D space. This would provide a new perspective of modeling and studying chromosomal 3D structures. For example, it would be interesting to cluster vertices based on network topology (with weights considered) and then compare the clusters in the networks with known genomic locations of topologically associating domains. The current definition of TADs is mostly based on 2D Hi-C enrichment. However, the network-clustering approach would be based on 3D structures although there is no need to construct the 3D structure.

Furthermore, since our inferred distances are already optimized, reconstructing a 3D structure from these distances becomes faster and less complicated. Also, two rounds of optimizations and the design of including FISH data in the first optimization (some of Eq. 4’s parameters are tuned by FISH data) make the reconstructed 3D structure more accurate and better fits the FISH observations (this is not the same as FISH data used to tune parameters in Eq. 4).

We notice that very limited chromosomal 3D structure reconstruction methods are evaluated using ChIA-PET. Therefore, we used two more measures to evaluate our inferred wish distances compared with those converted from α = 1/3. First, we found that when we only considered the number of Hi-C contacts in the range [12, 12.5] our inferred wish distances between beads within the same TAD are apparently smaller than those from different TADs, which better matches the property of TADs. Second, our inferred wish distances have a higher correlation with Xist transcript localization than those distances inferred from α = 1/3. To evaluate the 3D structures we inferred, we used another FISH data set; and the results show that our inferred 3D structures are more consistent with the new FISH data set than those generated by other two 3D-resconstruction methods PASTIS and ChromSDE with different α values.

## Conclusions

We developed a novel method to infer the wish distances between DNA bead-pairs from Hi-C contacts. Our inferred distances better fitted the definitions of TADs, FISH data, and the localization patterns of Xist transcripts compared to the distances generated by using a fixed parameter. High-resolution 3D structures of chromosomes were built based on the newly-inferred wish distances. The whole process has been implemented as a tool named HiCNet.

## Additional file

Additional file 1: Supplementary figures. **Figure S1.** (a) the converting function (α = 1/3) from Hi-C contacts to spatial distances; (b) the Hi-C contact distribution only considering two beads within 20 beads apart; (c) an illustration of triangle definition in HiCNet networks. **Figure S2.** The distribution of α values for the twenty chromosomes in mES. **Figure S3.** The distribution of Hi-C contacts between the beads with α parameters at top 5% and between beads with α parameters at bottom 5%. **Figure S4.** The distribution of Hi-C contacts between the beads with α parameters at top 20% and between beads with α parameters at bottom 20%. **Figure S5.** The Spearman correlations between α_ij_ values and corresponding Hi-C contacts *c*_*ij*_. Here we only use *c*_*ij*_ with |*i* - *j*| > 0.1*number of beads on a chromosome and *c*_*ij*_ ≠ 0. **Figure S6.** The plot of chromosome 9’s Hi-C contacts against inferred wish distances. The blue lines indicate the inverse relationship between Hi-C contacts (<= 50) and inferred wish distances. **Figure S7.** The heatmap of the Euclidean distances parsed from the 40 kb resolution 3D structure of X-chromosome generated by PASTIS with α equal to 0.35. The heatmap is in 500 kb, i.e., we average the distances of 40 kb beads into 500 kb. **Figure S8.** The heatmap of the Euclidean distances parsed from the 40 kb resolution 3D structure of X-chromosome generated by ChromSDE with α equal to 0.35. The heatmap is in 500 kb, i.e., we average the distances of 40 kb beads into 500 kb. (DOCX 1532 kb)

## Abbreviations

3D: Three-dimensional
FISH: Fluorescence in situ hybridization
HiCNet: Hi-C network
TAD: Topologically associating domain
