# Supplementary material for: Reconstructing high-resolution chromosome three-dimensional structures by Hi-C complex networks
Source: BMC Bioinformatics. 2018 Dec 28;19(Suppl 17):496. doi: 10.1186/s12859-018-2464-z (PMC6309071; doi:10.1186/s12859-018-2464-z)
Supplement: Supplementary file 1 — Supplementary figures. Figure S1. (a) the converting function (α = 1/3) from Hi-C contacts to spatial distances; (b) the Hi-C contact distribution only considering two beads within 20 beads apart; (c) an illustration of triangle definition in HiCNet networks. Figure S2. The distribution of α values for the twenty chromosomes in mES. Figure S3. The distribution of Hi-C contacts between the beads with α parameters at top 5% and between beads with α parameters at bottom 5%. Figure S4. The distribution of Hi-C contacts between the beads with α parameters at top 20% and between beads with α parameters at bottom 20%. Figure S5. The Spearman correlations between αij values and corresponding Hi-C contacts cij. Here we only use cij with |i - j| > 0.1*number of beads on a chromosome and cij ≠ 0. Figure S6. The plot of chromosome 9’s Hi-C contacts against inferred wish distances. The blue lines indicate the inverse relationship between Hi-C contacts (<= 50) and inferred wish distances. Figure S7. The heatmap of the Euclidean distances parsed from the 40 kb resolution 3D structure of X-chromosome generated by PASTIS with α equal to 0.35. The heatmap is in 500 kb, i.e., we average the distances of 40 kb beads into 500 kb. Figure S8. The heatmap of the Euclidean distances parsed from the 40 kb resolution 3D structure of X-chromosome generated by ChromSDE with α equal to 0.35. The heatmap is in 500 kb, i.e., we average the distances of 40 kb beads into 500 kb. (DOCX 1532 kb) [file 12859_2018_2464_MOESM1_ESM.docx]

**Reconstructing high-resolution chromosome three-dimensional structures by Hi-C complex networks**

Additional file 1

The widely used converting function from Hi-C contacts to spatial distances is shown in Figure S1a. From this figure, we can observe that when Hi-C contacts are larger than 10, the converted distances vary more slightly than those from Hi-C contacts less than 10. However, there are lots of Hi-C contacts larger than 10 (Figure S1b). Therefore, using the converting function (α=1/3) for all Hi-C contacts will lose the variances of distances for those Hi-C contacts larger than 10, which may have a negative effect on predicting three-dimensional structures of chromatins.


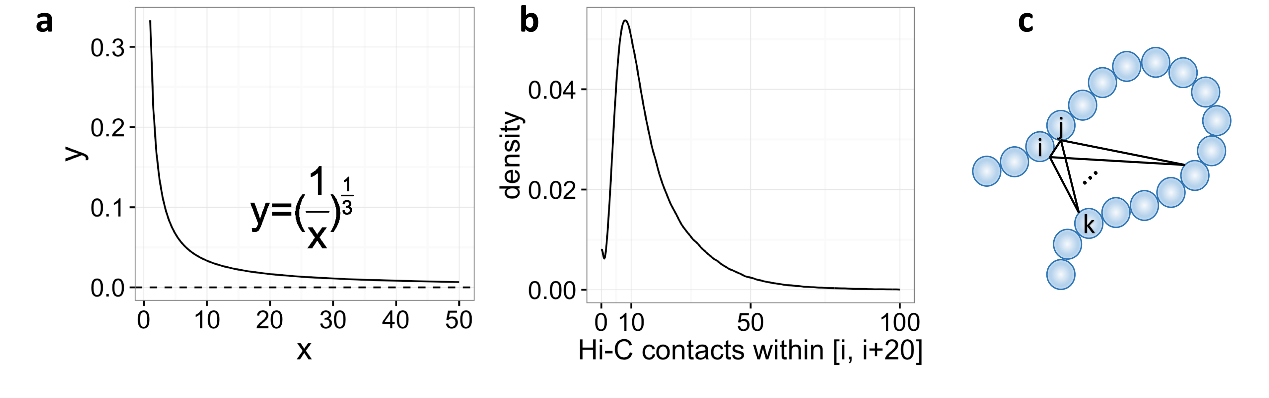


Figure S1. (a) the converting function (α=1/3) from Hi-C contacts to spatial distances; (b) the Hi-C contact distribution only considering two beads within 20 beads apart; (c) an illustration of triangle definition in HiCNet networks.


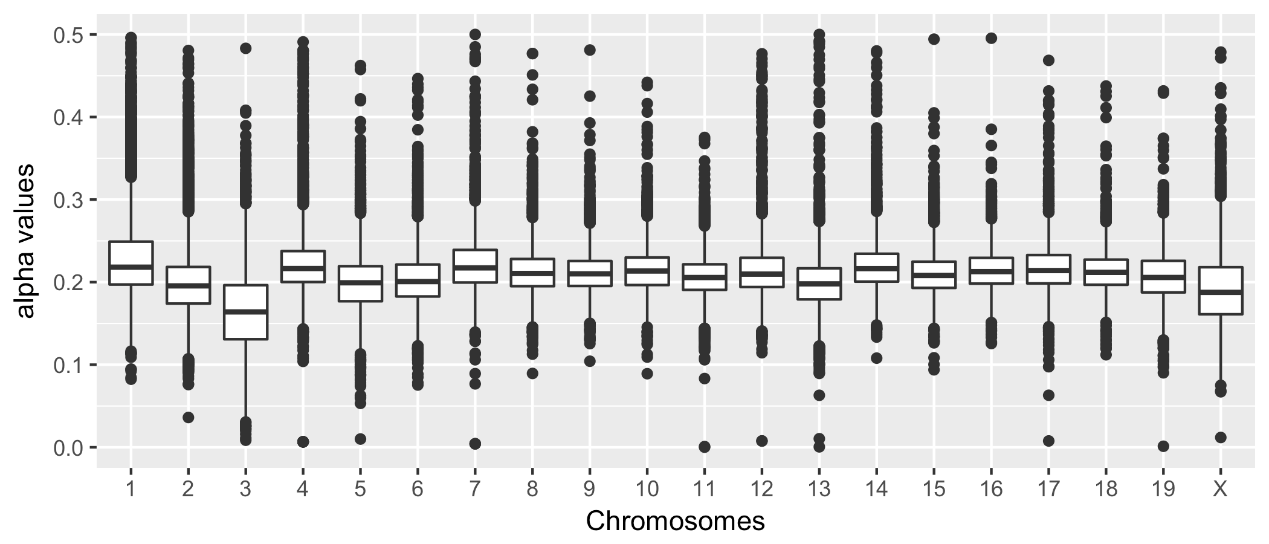


Figure S2. The distribution of α values for the twenty chromosomes in mES.


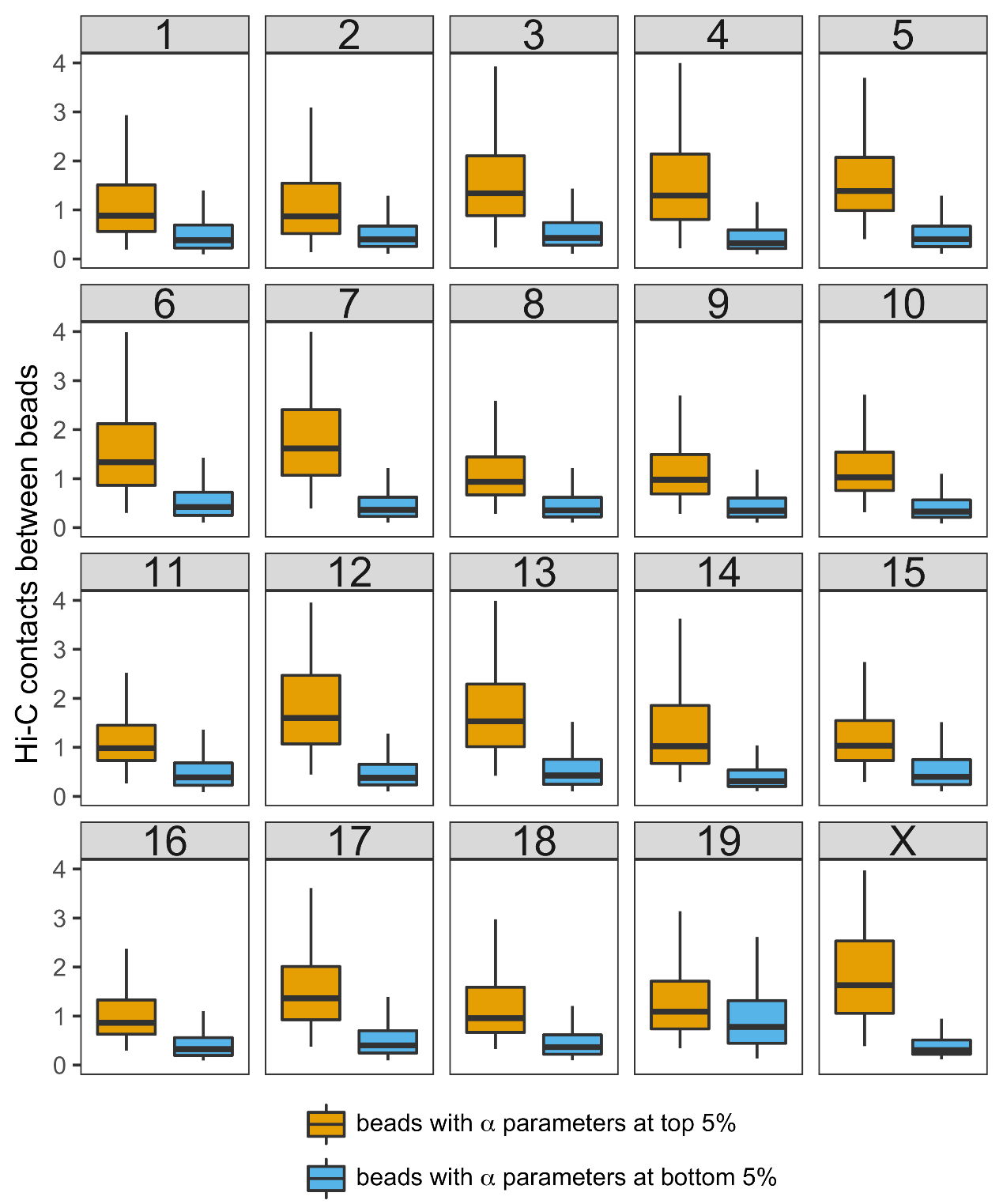


Figure S3. The distribution of Hi-C contacts between the beads with α parameters at top 5% and between beads with α parameters at bottom 5%.


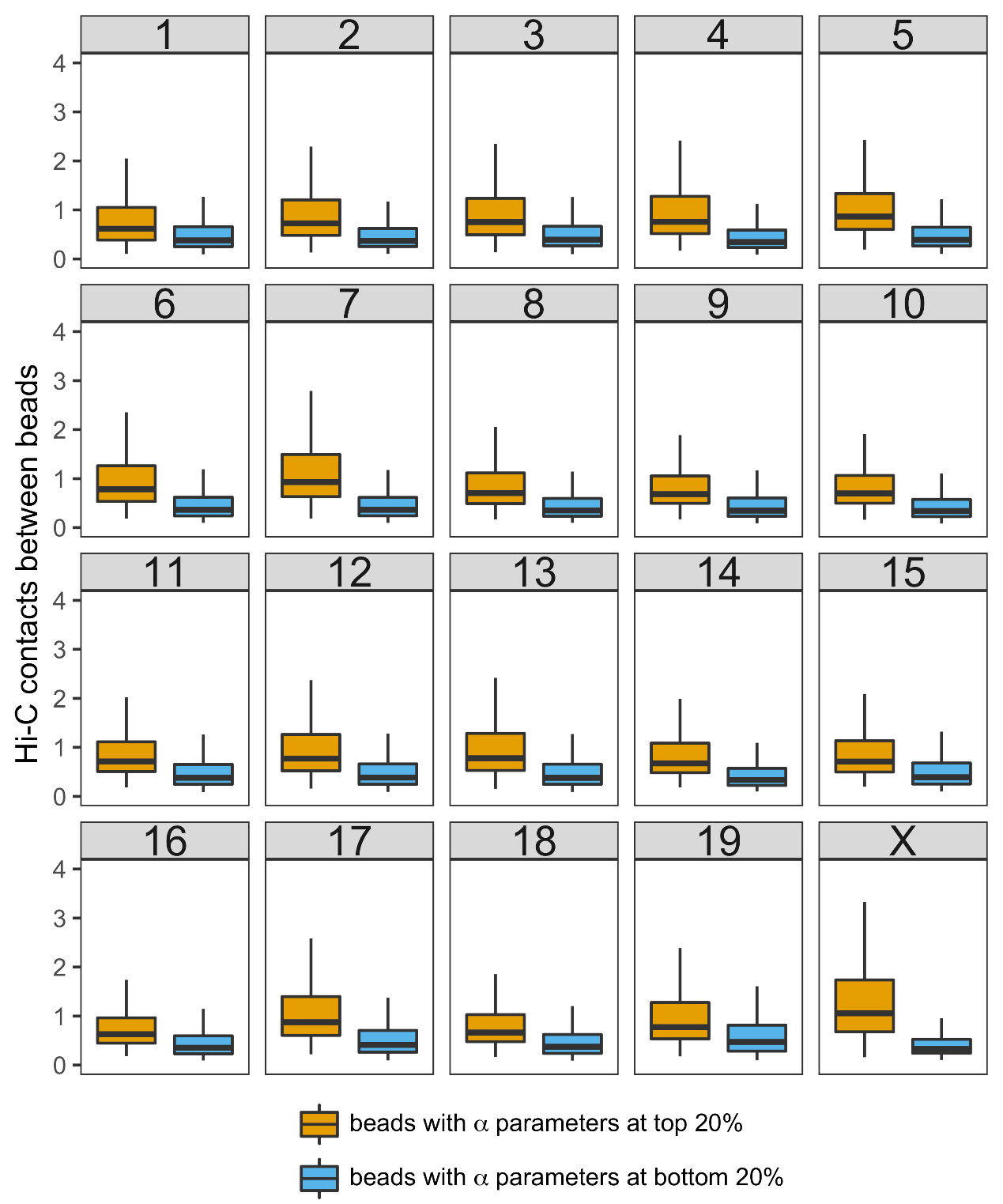


Figure S4. The distribution of Hi-C contacts between the beads with α parameters at top 20% and between beads with α parameters at bottom 20%.


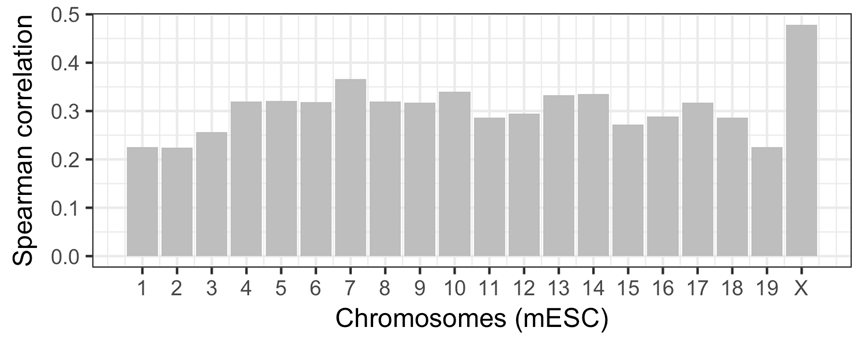


Figure S5. The Spearman correlations between α_ij_ values and corresponding Hi-C contacts *c_ij_*. Here we only use *c_ij_* with |*i* - *j*| > 0.1*number of beads on a chromosome and *c_ij_* ≠ 0.


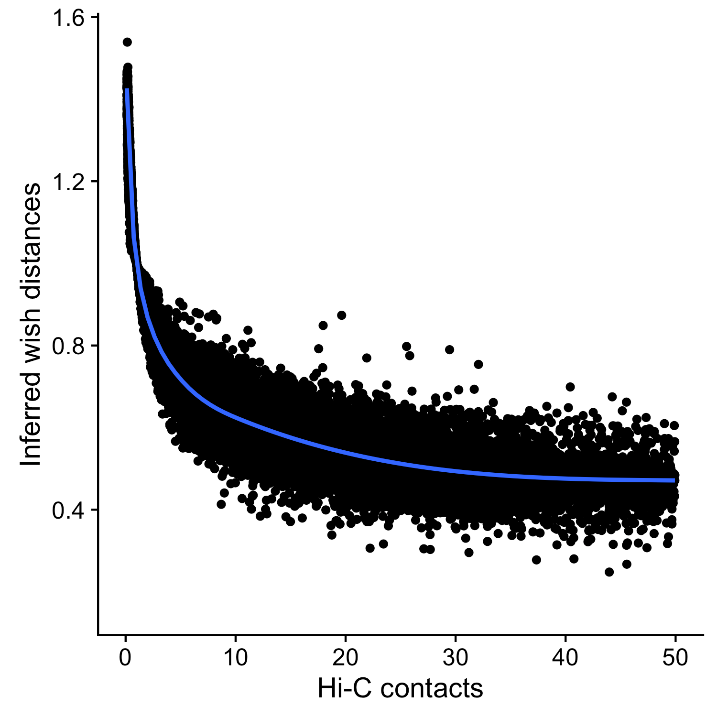


Figure S6. The plot of chromosome 9’s Hi-C contacts against inferred wish distances. The blue lines indicate the inverse relationship between Hi-C contacts (<= 50) and inferred wish distances.


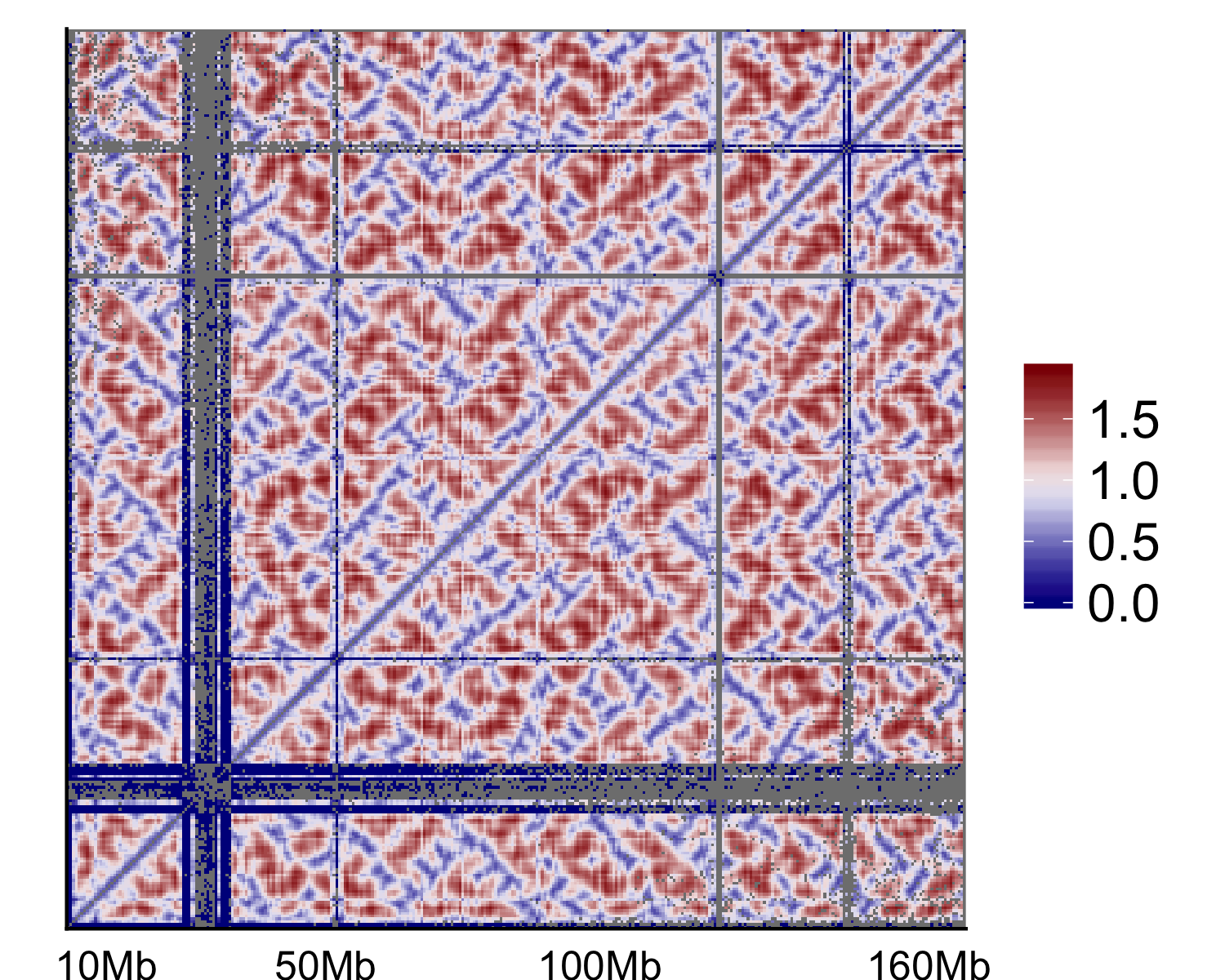


Figure S7. The heatmap of the Euclidean distances parsed from the 40 kb resolution 3D structure of X-chromosome generated by PASTIS with α equal to 0.35. The heatmap is in 500 kb, i.e., we average the distances of 40 kb beads into 500 kb.


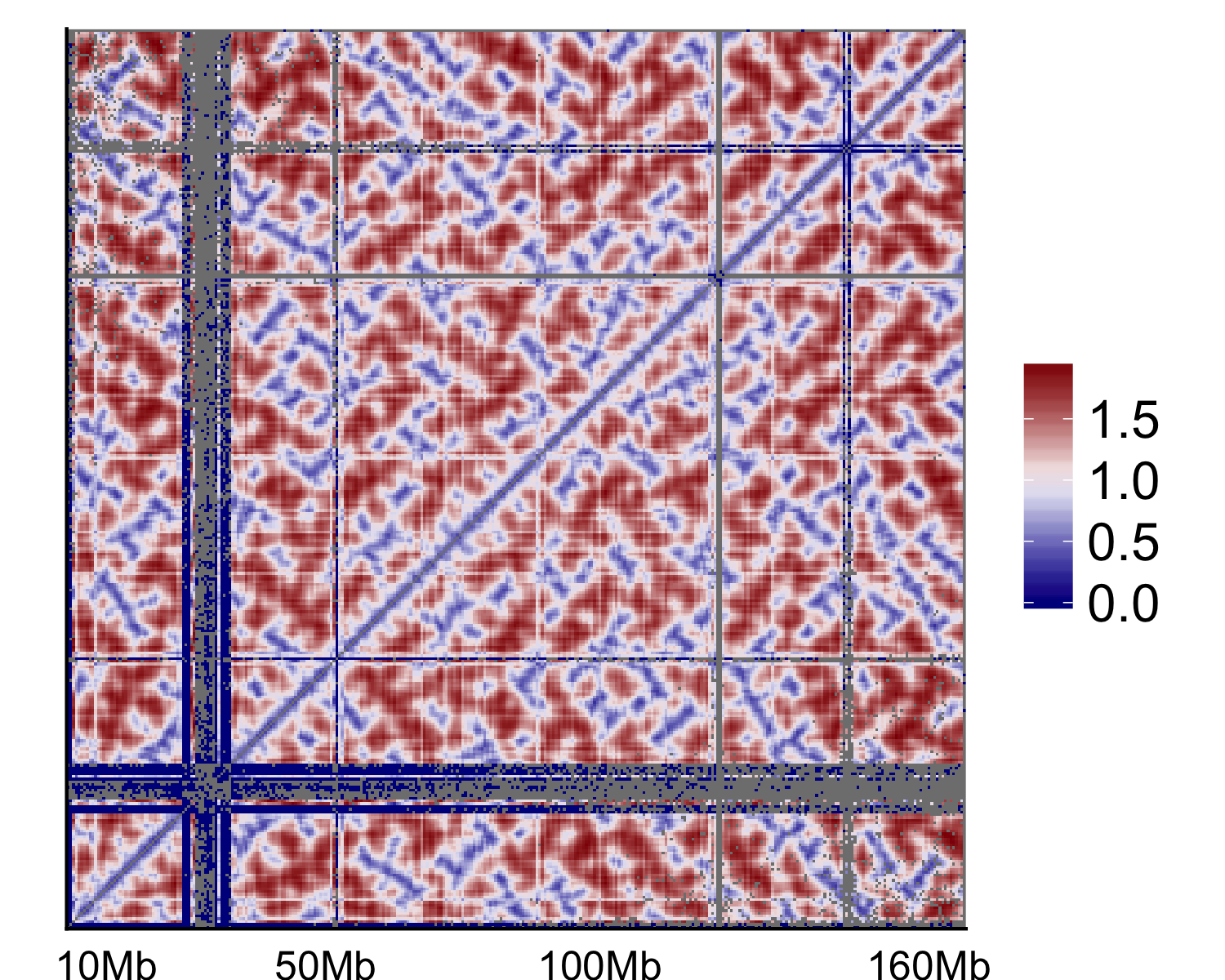


Figure S8. The heatmap of the Euclidean distances parsed from the 40 kb resolution 3D structure of X-chromosome generated by ChromSDE with α equal to 0.35. The heatmap is in 500 kb, i.e., we average the distances of 40 kb beads into 500 kb.
